# An eight-mRNA signature predicts the prognosis of patients with bladder urothelial carcinoma

**DOI:** 10.7717/peerj.7836

**Published:** 2019-10-22

**Authors:** Rui Zhu, Xin Yang, Wenna Guo, Xin-Jian Xu, Liucun Zhu

**Affiliations:** 1 Department of Mathematics, Shanghai University, Shanghai, China; 2 School of Life Sciences, Shanghai University, Shanghai, China; 3 School of Life Sciences, Zhengzhou University, Zhengzhou, Henan, China

**Keywords:** Bladder urothelial carcinoma, Prognosis, Biomarker, Risk-stratification

## Abstract

**Background:**

Bladder cancer is one of the most common cancers, and its histopathological type is mainly bladder urothelial carcinoma, accounting for about 90%. The prognostic biomarkers of bladder cancer are classified into clinical features biomarkers and molecular biomarkers. Nevertheless, due to the existence of individual specificity, patients with similar pathological characteristics still have great differences in the risk of disease recurrence. Therefore, it is often inaccurate to predict the survival status of patients based on clinical characteristic biomarkers, and a prognostic molecular biomarker that can grade the risk of bladder cancer patients is needed.

**Methods:**

A total of three bladder urothelial carcinoma datasets were used in this study from the Cancer Genome Atlas database and Gene Expression Omnibus database. In order to avoid overfitting, all samples were randomly divided into one training set and three validation sets, which were used to construct and test the prognostic biomarker model of bladder urothelial carcinoma. Univariate and multivariate Cox regression were used to screen candidate mRNAs and construct prognostic biomarkers model. Kaplan–Meier survival analysis and the receiver operating characteristic (ROC) curve were used to evaluate the predictive performance of the model.

**Results:**

A prognostic biomarker model of bladder urothelial carcinoma combining with eight mRNA was constructed. Kaplan–Meier analyses indicated that a significant difference in the survival time of patients between the high-risk and the low-risk group. The area under the ROC curve were 0.632 (95% confidence interval (CI) [0.541–0.723]), 0.693 (95% CI [0.601–0.784]) and 0.686 (95% CI [0.540–0.831]) when the model was used to predict the patient’s survival time in three validation datasets. The model showed high accuracy and applicability.

## Introduction

Bladder cancer, which is one of the most common cancers, is a malignant tumor that occurs on the mucous membrane of the bladder. A total of 70% newly diagnosed patients present with superficial tumor, which are usually not life-threatening, but are prone to recurrence after operation. The remaining 30% patients with muscle infiltration have a higher risk of distant metastasis and death. The most frequent histopathological type of bladder cancer is mainly bladder urothelial carcinoma, which accounts for about 90% of cases (Schwartz et al., 2003). The 5-year survival rate for patients with distal metastasis is less than 30% (Karl et al., 2009; Ugurlu et al., 2015; Zehnder et al., 2014), which poses serious threat to human life and health (Torre et al., 2015).

In the treatment of bladder cancer, surgical resection is still the first choice for radical therapy. The 5-year survival rate for some patients can be greatly improved, but others still develop recurrence, while the overall 5-year survival rate is still low (Smith et al., 2013), suggesting that surgical excision is not suitable for all patients. Therefore, a prognostic marker for risk classification for patients with bladder cancer is needed, as well as further consideration of other or adjuvant treatments that should be given to patients who are not suitable for surgical treatment. These would help to make a more accurate assessment of bladder cancer and to achieve a more targeted effective treatment (Peng et al., 2017).

The prognostic biomarkers of bladder cancer are classified into clinical features biomarkers and molecular biomarkers. There are a number of studies on the clinical features biomarkers of bladder cancer, such as tumor size, character, deteriorating state of tumor, number of lymph node metastases, clinical staging and classification (Riester et al., 2012; Sylvester et al., 2006). Nevertheless, due to the existence of individual specificity, the recurrence risk of patients with similar pathological characteristics is still very different. Therefore, when predicting patient’s survival state according to their clinical feathers, the judgment is often inaccurate (Avgeris et al., 2015). Recently, it has been found that the expression level of some molecules, especially some mRNA (He et al., 2015; Shi et al., 2015), can be used to predict the survival time of cancer patients. For example, Zeng et al. (2017) have found that the expression levels of ANLN in tumor tissue are significantly correlated with the survival time of patients with bladder cancer. Tokas et al. (2017) have demonstrated the dysregulation of KLK13 in bladder tumors through clinical studies, and emphasized that KLK13 is a promising marker for improving the prognosis of bladder urothelial carcinoma (BLCA) patients. In addition, Liu & Zhang (2017) have pointed out that lncRNA *PVT1* is highly expressed in the tumor tissue that produces drug resistance, and its silence is closely related to the proliferation, invasion and resistance of tumor cells, which can be used as an independent prognostic biomarker for bladder cancer patients. Hu et al. (2017) have analyzed the miRNA expression profile of 399 bladder cancer patients in The Cancer Genome Atlas (TCGA) database, and they found that 19 miRNA were significantly related to the survival time of patients, while three of them could be used independently as the prognostic biomarkers.

Compared with the single molecular biomarker mentioned above, the combinatorial prognostic molecular biomarker showed significant advantages in the risk classification and prognostic analysis of patients. Furthermore, it revealed to have high accuracy, strong sensitivity and wide applicability (Guo et al., 2016). So far, there were several studies on bladder cancer’s the model of molecular combinatorial biomarkers. For example, Liu et al. (2017) have established a 3-mRNA risk staging model for BLCA patients that was independent of and superior to other clinical information. Bao, Zhang & Dong (2017) have constructed a 4-lncRNA prognostic molecular biomarker model by analyzing the lncRNA expression profile of 234 patients in the TCGA database. In addition, Sapre et al. (2016) have screened out a 6-miRNA prognostic model by analyzing the miRNA expression profile of 131 patients. Yet, these existing studies on molecular combinatorial biomarker models would have some limitations, such as relatively small sample cohorts, lacking the cross-platform validating datasets or narrow focusing on patient specimens with specific clinical features. Moreover, the predictive accuracy and reproducibility of known prognostic markers of BLCA is limited.

The purpose of this study was to analyze the expression level of all mRNA in bladder urothelial carcinoma tissue, to screen out candidate mRNAs and to construct a combinatorial prognostic biomarkers model by using univariate and multivariate Cox regression. Finally, the performance of the model was tested by receiver operating characteristic (ROC) analysis in two cross-platform datasets, a precise and stable biomarker model combining with mRNA was constructed to predict the prognosis of bladder cancer patients. The model can be used to improve the prognosis of patients and to provide individualized treatment options for patients.

## Materials and Methods

### Data source

A total of three datasets were used in this study, which were the bladder urothelial carcinoma dataset (TCGA-BLCA) from TCGA database (https://cancergenome.nih.gov/) (Tomczak, Czerwinska & Wiznerowicz, 2015) and cross-platform dataset (GSE13507 and GSE31684) from The National Center for Biotechnology Information (NCBI) gene expression omnibus (GEO) dataset (https://www.ncbi.nlm.nih.gov/geo/query/acc.cgi). There were 412 samples of bladder urothelial carcinoma in the TCGA-BLCA, among which, there were no information about survival time for two sample and no information about the expression level of mRNA for four samples. After excluding these six samples, the retained 406 samples were used as the basic data for analysis. Among these 406 samples, 19 samples had mRNA expression data of both cancerous and paracancerous tissues, so we extracted these 19 samples for differential expression analysis. Then, in order to avoid overfitting, the remaining 387 samples were randomly divided into independent the training set (194 samples) and the validation set (193 samples), which were used to construct and test the prognostic biomarker model of bladder urothelial carcinoma, respectively. The detailed information of BLCA patients were listed in Table 1. In particular, the GSE13507 dataset contains 256 samples, of which 91 samples did not contain survival information. We removed these data from the study. The remaining 165 samples, together with the GSE31684 dataset which contains 65 samples of bladder urothelial carcinoma, were used to further evaluate the applicability of the biomarker model.

**Table 1:** Clinicopathological characteristics of BLCA patients from TCGA database.

Characteristics	Groups	Patient					
		Training set		Validation set		Sum	
		Amount	%	Amount	%	Amount	%
Age at diagnosis							
	>60	145	74.74	140	72.54	285	73.64
	≤60	49	25.26	53	27.46	102	26.36
Gender							
	Male	141	72.68	148	76.68	289	74.68
	Female	53	27.32	45	23.32	98	25.32
Tumor stage							
	Stage I	1	0.52	1	0.52	2	0.52
	Stage II	63	32.47	62	32.12	125	32.30
	Stage III	64	32.99	69	35.75	133	34.36
	Stage IV	64	32.99	61	31.61	125	32.30
	Others/unknown	2	1.03	0	0.00	2	0.52
Clinical stage							
	Low grade	12	6.19	8	4.15	20	5.17
	High grade	179	92.27	185	95.85	364	94.06
	Others/unknown	3	1.54	0	0.00	3	0.77
Survival status							
	Survive	116	59.79	105	54.40	221	57.11
	Death	78	40.21	88	45.60	166	42.89

### Statistical analysis and calculation methods

We used R language to analyze the univariate Cox regression, the multivariate Cox regression and the ROC curve, and judged the predictive performance of the model according to the areas under the ROC curve (AUC). The univariate Cox proportional hazard analysis was first performed in the training dataset to identify mRNAs that significantly (*P* < 0.05) correlated with patient survival as candidate markers. Then, the multivariate Cox regression analysis was carried out to further screen the model that associated with patients’ survival time, we exhaustively selected two, three … eight mRNAs from the candidate markers as covariates and constructed models. Subsequently, the AUC was calculated to measure and compare the models, and the model with the highest AUC was eventually selected out. In the study, the gene expression values from TCGA were calculated as Fragments Per Kilobase of transcript per Million mapped reads (FPKM) values (Howe et al., 2011; Leek, 2014), and FPKM-UQ values (Bullard et al., 2010; Shahriyari, 2019) that is a modified version of the FPKM formula were used. Then log2(FPKM-UQ+1) was used for normalizing the gene expression values. Furthermore, the gene expression values from GEO database were directly used for Cox regression analysis. In order to improve the operation efficiency, we used Perl language to carry out parallel calculation, quickly obtained all feasible biomarker models and evaluated their comprehensive performance.

## Results

### Construction of mRNA prognostic biomarker model

Each sample data contained the expression levels of 60,483 mRNA. In order to make the prognostic biomarkers applicable on multiple platforms, we firstly screened out the genes that could be detected by using eight common probe chips (AFFY HG U133 Plus 2 probe, AGILENT SurePrint G3 GE 8x60k v2 probe, AGILENT WholeGenome 4x44k v2 probe, AGILENT GPL6848 probe, ILLUMINA HumanHT 12 v4 probe, ILLUMINA HumanRef 8 v3 probe, ILLUMINA HumanWG 6 v3 probe, ILLUMINA Human-6 v2 probe), and 11,849 mRNA were obtained. Then, in order to find out the prognostic biomarker suitable for most patients, we deleted the mRNA with expression level less than 50%, thus obtaining 11,111 mRNA. Next, using the previously extracted samples with RNA expression data of both cancerous and paracancerous tissues, the paired two-sample signature rank test was performed to select 5,667 mRNA with significant differences in the expression levels between the two tissues (*P* ≤ 0.05). Moreover, the univariate Cox regression was carried out based on patient’s survival time and the expression level of 5,667 mRNA. The Wald test was carried out to retain 940 mRNA as the candidates for the construction of model next; for these mRNA, the *P*-values were less than 0.05 and they were significantly related to survival time.

The possible combinations of all one to eight genes were constructed using multivariate Cox regression. By comparing of the area under the ROC curve (AUC), the highest AUC of the prognostic biomarker model was finalized. Finally, a following prognostic biomarker model containing eight mRNA was identified:

$$\eqalign{
  & {\rm{Risk score}} = \left( {0.25457 \times {{\rm{E}}_{{\rm{ENSG00000011426}}}}} \right) - \left( {0.25465 \times {{\rm{E}}_{{\rm{ENSG00000100285}}}}} \right)  \cr 
  & \quad \quad \quad \quad \quad \;\, + \left( {0.08186 \times {{\rm{E}}_{{\rm{ENSG00000105492}}}}} \right) + \left( {0.38131 \times {{\rm{E}}_{{\rm{ENSG00000106348}}}}} \right)  \cr 
  & \quad \quad \quad \quad \quad \;\, + \left( {0.15089 \times {{\rm{E}}_{{\rm{ENSG00000135218}}}}} \right) - \left( {0.11896 \times {{\rm{E}}_{{\rm{ENSG00000140093}}}}} \right)  \cr 
  & \quad \quad \quad \quad \quad \;\, + \left( {0.25721 \times {{\rm{E}}_{{\rm{ENSG00000163132}}}}} \right) + \left( {0.79316 \times {{\rm{E}}_{{\rm{ENSG00000183576}}}}} \right) \cr} $$

E_ENSG00000011426_, E_ENSG00000100285_, E_ENSG00000105492_, E_ENSG00000106348_, E_ENSG00000135218_, E_ENSG00000140093_, E_ENSG00000163132_, E_ENSG00000183576_ were accordingly referred to the mRNA expression levels of ENSG00000011426, ENSG00000100285, ENSG00000105492, ENSG00000106348, ENSG00000135218, ENSG00000140093, ENSG00000163132, ENSG00000183576. The risk score represented the death risk for BLCA patients. As shown in the model, the expression level of ENSG00000100285 and ENSG00000140093 were negatively correlated with BLCA patient survival time, while the expression level of other mRNA were positively correlated with the BLCA patient survival time.

According to Genome Reference Consortium Human Build 38 patch release 12 (GRCh38.p12), the chromosome locations of the eight mRNAs were described below in Table 2.

**Table 2:** Information of eight mRNAs in the optimal prognostic biomarker model obtained via multivariate Cox regression.

Ensembl Id	Chr.	Coordinate	Coefficient^a^	*P*-value^b^
ENSG00000011426	Chr7	36,389,803–36,453,791	0.25457	0.0303
ENSG00000100285	Chr22	29,480,192–29,491,290	−0.25465	0.0465
ENSG00000105492	Chr19	51,517,819–51,531,856	0.08186	0.000386
ENSG00000106348	Chr7	128,392,277–128,409,989	0.38131	0.0236
ENSG00000135218	Chr7	80,602,188–80,679,277	0.15089	0.0231
ENSG00000140093	Chr14	94,280,460–94,293,353	−0.11896	0.00391
ENSG00000163132	Chr4	4,859,665–4,863,936	0.25721	0.024
ENSG00000183576	Chr14	99,397,746–99,486,458	0.79316	0.00476

**Notes:**

a The coefficient value of the gene in the prognostic model derived from the multivariate Cox regression analysis.

b The Wald test *P* value in the multivariate Cox regression analysis.

### Performance evaluation of mRNA prognostic biomarker model in TCGA validation set and GEO validation set

**Figure 1:**
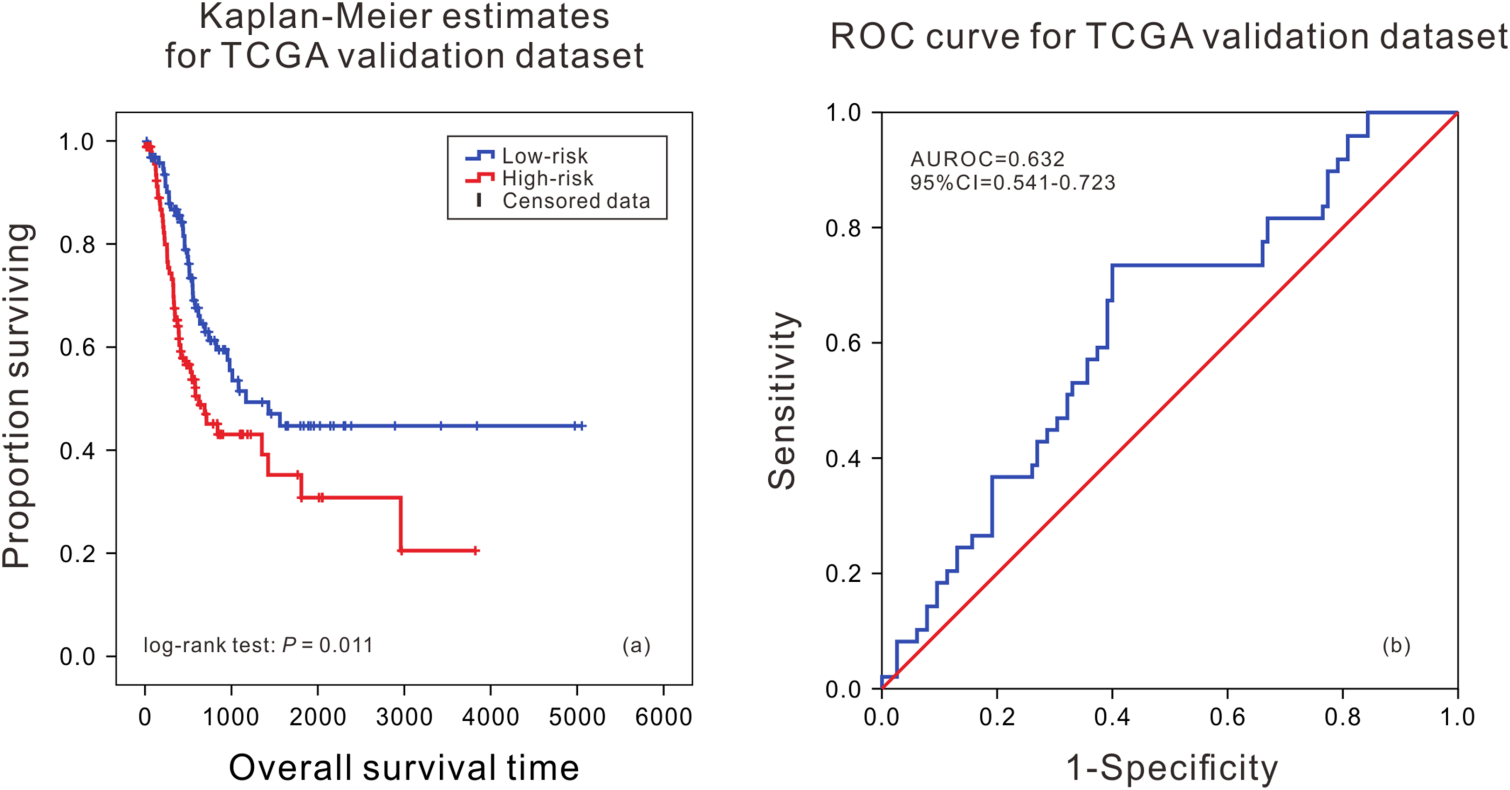
Kaplan–Meier analysis and ROC curve from the TCGA validation dataset. Kaplan–Meier analysis of survival difference between high and low risk BLCA patients from the TCGA validation dataset (A); ROC curve of survival prediction by mRNA prognostic biomarker from the TCGA validation dataset (B).

According to the median of risk score calculated by the model, the patients were divided into the high death risk group and the low death risk group. In the training dataset, the mean survival time of patients in the high death risk group was 753 days, which was 223 days less than that of the low death risk group. In the validation dataset, the mean survival time of patients in the high death risk group was 632 days, which was 292 days less than that of the low death risk group. Next, the Kaplan–Meier curve and time series analysis were used to compare the survival time of patients between the two groups, and the results showed that there was a significant difference in the survival time of patients between the two groups in the TCGA validation dataset (*P* = 0.011) (Fig. 1A).This shows that the mRNA prognostic biomarker model constructed in the training dataset was accurate and reproducible, and can accurately predict the risk of death in BLCA patients. At the same time, the AUC values of the ROC curves were calculated by time-dependent ROC analysis using the median survival time of patients as the cutoff point, the AUC was 0.632 (95% CI [0.541–0.723]) in the validation dataset (Fig. 1B), reflecting that the prognostic marker model composed of eight mRNAs had high sensitivity and specificity in predicting patients’ survival status.

**Figure 2:**
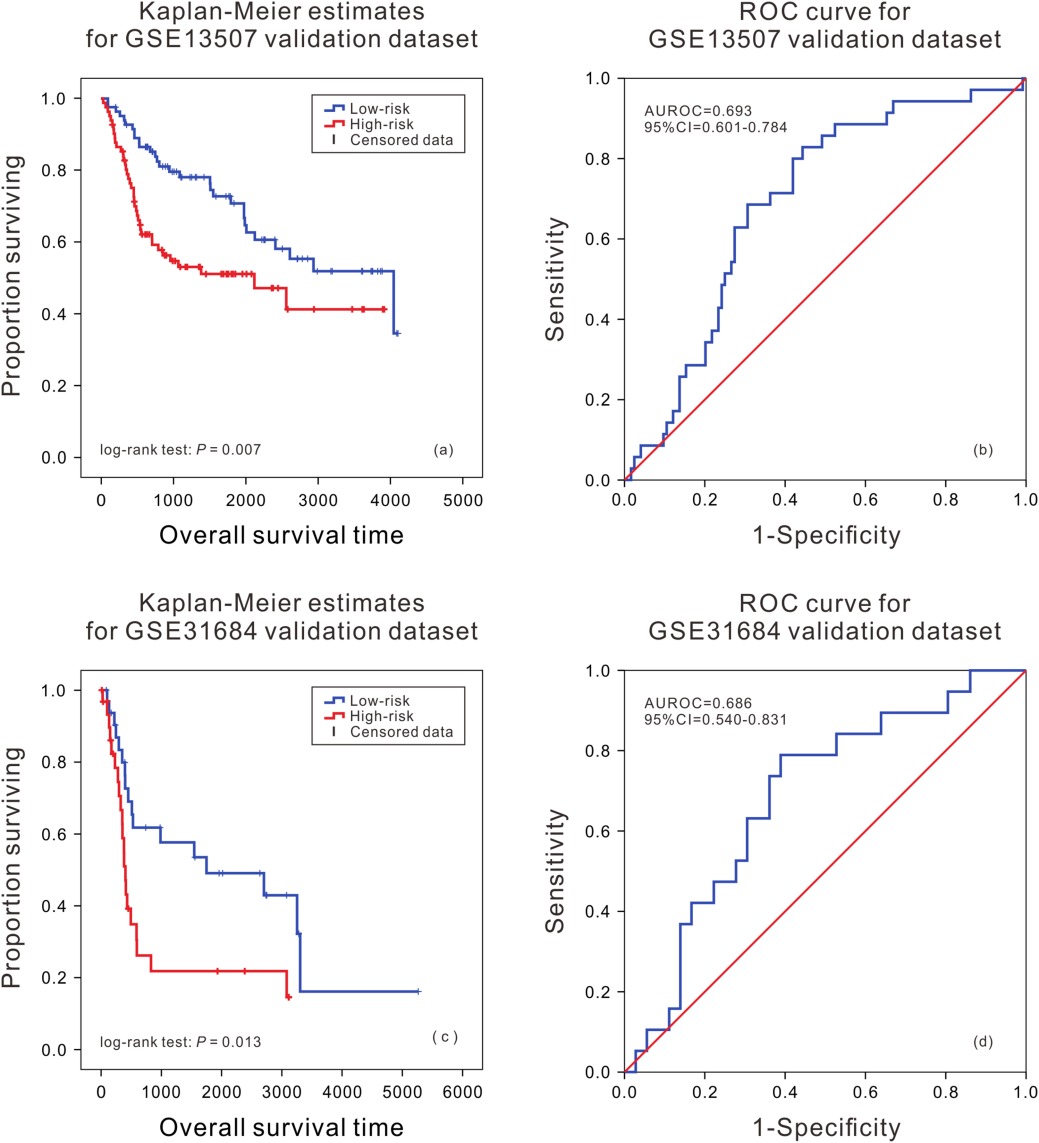
Kaplan–Meier analysis and ROC curve from two GEO validation datasets. Kaplan–Meier analysis of survival difference between high and low risk BLCA patients from the GSE13507 (A) and GSE31684 validation dataset (C); ROC curve of survival prediction by mRNA prognostic biomarker from the GSE13507 (B) and GSE31684 validation dataset (D).

In order to further evaluate the applicability of the mRNA prognostic biomarker model, two GEO datasets were used as the cross-platform dataset to test the model. The patients in the GEO validation dataset were divided into two groups. There was a significant difference in the survival time of patients between the high death risk group and the low death risk group (*P* = 0.007, *P* = 0.013) (Figs. 2A and 2C), and the area under the ROC was 0.693 (95% CI [0.601–0.784]) and 0.686 (95% CI [0.540–0.831]) (Figs. 2B and 2D) in GSE13507 and GSE31684, respectively.

### Performance evaluation of mRNA prognostic biomarker model at different clinical features

Due to patient’ gender, clinical stage and age could be used as prognostic factors, and they affected the occurrence and development of cancer to some extent. The bladder urothelial carcinoma patients in the TCGA were regrouped according to these three clinical features to further verify the practicability of the mRNA prognostic biomarker model (Table 3). By plotting the ROC curves of the model in each regrouped groups, it was found that the AUCs were all greater than 0.7. Kaplan–Meier analysis also showed a significant difference for the survival time between the high and low risk groups (*P* < 0.01) in all groups except the older and stage I & II group. The results showed that the model was applicable to patients of different genders and those with relatively high risk of stage III, stage IV and older bladder urothelial carcinoma patients (Figs. S1–S3 and S5).

**Table 3:** Kaplan–Meier and ROC results of different regrouping methods.

Regrouping factor	Groups	Amount	Kaplan–Meier *P*-value	AUC	AUC 95% confidence interval	Median abundance of the genes
Gender						
	Male	289	<0.001	0.78	[0.711–0.849]	29.361
	Female	98	0.004	0.703	[0.575–0.830]	29.527
Tumor stage						
	Stage I & II	127	0.164	0.729	[0.591–0.867]	29.023
	Stage III	133	<0.001	0.792	[0.694–0.890]	29.442
	Stage IV	125	0.001	0.733	[0.633–0.833]	29.705
Age						
	≤60	102	0.094	0.78	[0.711–0.849]	29.214
	>60	285	<0.001	0.757	[0.689–0.825]	29.478

### Comparison of the prognostic biomarker model with other known prognostic biomarkers

**Figure 3:**
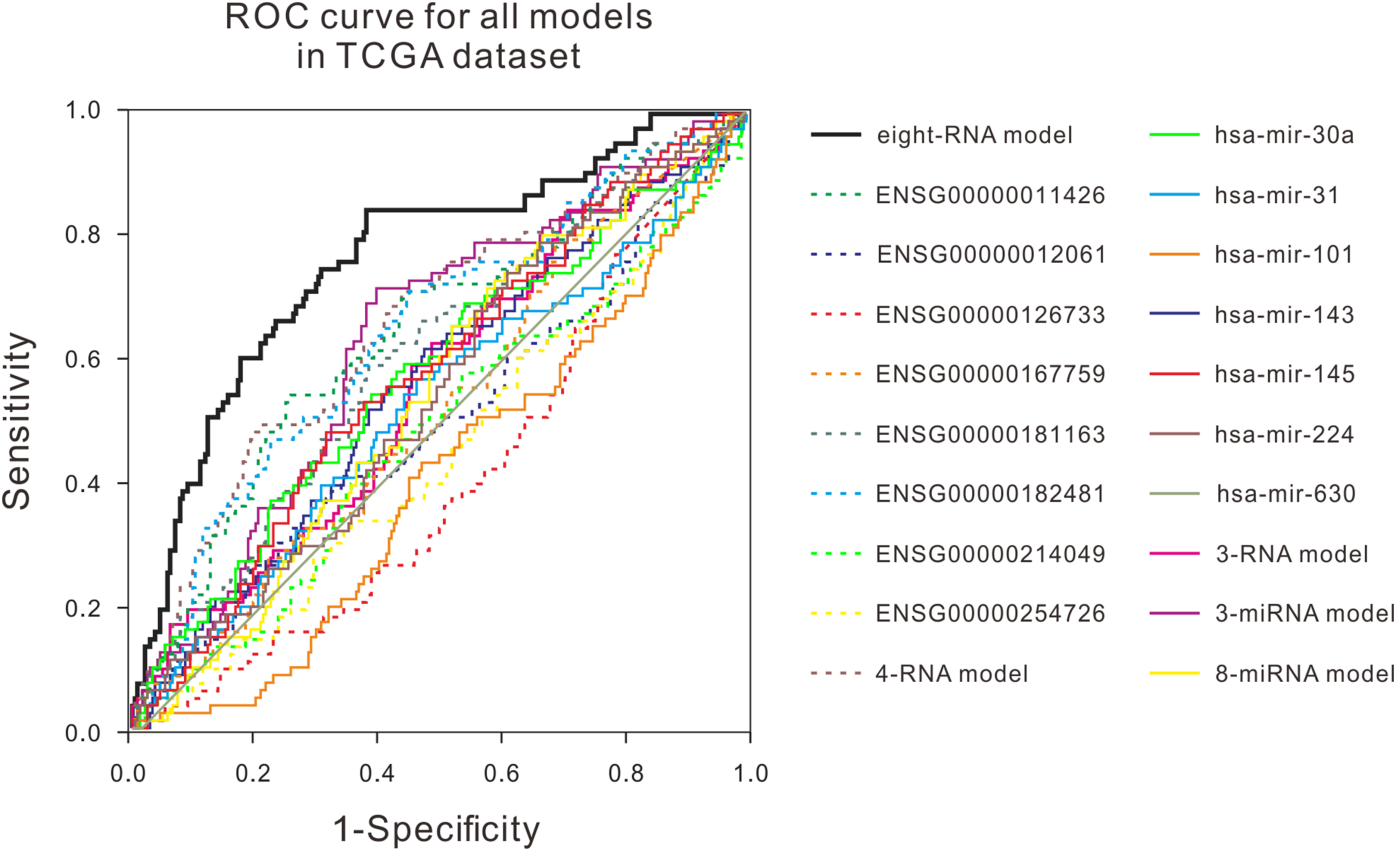
ROC curves were compared with other known prognostic biomarkers in the TCGA dataset. In TCGA dataset, the ROC curve of prediction property of RNA prognostic biomarker combination for BLCA in comparison with other known prognostic biomarkers.

Many studies have shown that a variety of molecules can be used as prognostic biomarkers for bladder urothelial carcinoma. In order to test the predictive ability of the mRNA prognostic biomarker model constructed in this study, the combination of molecular prognostic biomarkers, and the ROC analysis was used to compare the performance prediction in the TCGA dataset and two GEO datasets (Fig. 3; Fig. S4). For other known prognostic markers, if the prognostic models of the biomarker was available in the previous research, the risk scores of patients in our validation dataset would be calculated using their models, then the AUCs were obtained, just as the analysis for our eight-RNA model. Nevertheless, if the biomarker was single gene or miRNA, the ROC analysis was performed directly with their expression values to verify their predictive performance in our validation datasets. As shown in the figures, the AUC of our combination was generally larger than other markers in the three datasets. The obtained results showed that the mRNA prognostic biomarker model was more accurate than other prognostic biomarkers or combinations in predicting patient’s survival status.

## Discussion

In the current study, a prognostic biomarker model of bladder urothelial carcinoma combining with eight mRNA was constructed. Through Kaplan–Meier and ROC analysis, it is shown that the model has high accuracy in the TCGA dataset, and an excellent prognostic performance in two cross-platform GEO datasets, which fully proving the applicability of the mRNA prognostic biomarkers. In order to reveal the effectiveness of the model, we further evaluated the predictive performance of the model at different clinical features in the TCGA dataset. The model had very high prediction accuracy, especially for high-risk patients with high clinical stage and patients over 60 years old. Finally, to reflect the overall predictive advantage of the prognostic biomarker model, we compared the predictive performance of our model with the molecular prognostic biomarkers found by other studies in the TCGA dataset and GEO dataset. The results showed that the mRNA prognostic biomarker combination not only used multiple markers to comprehensively evaluate the survival risk of patients, but also had significant advantages over other markers in predictive performance and reproducibility, which are expected to be very useful for the selection of clinical treatment strategies in the future.

In the model constructed in this study, three genes have been reported to be closely related to tumorigenesis, cell cycle and other carcinogenesis. ANLN (ENSG00000011426) encodes an actin-binding protein that plays a role in cell growth and migration, and in cytokinesis (Zhou et al., 2015). The expression level of ANLN gene is closely related to the invasiveness of tumors, and it is highly expressed in various types of malignant tumors such as breast cancer, colon cancer, liver cancer and lung cancer (Hall et al., 2005; Piekny & Maddox, 2010). CD36 (ENSG00000135218) is a scavenger receptor protein, which is an important regulator of lipid metabolism and innate immunity and mediates the uptake of fatty acids in different types of cells. By inhibiting CD36, the occurrence of lymph node metastasis can be blocked, and the presence of CD36 positive cells is associated with the recurrence of metastasis of malignant tumors in different systems, suggesting that CD36 may play an important role in the recurrence of tumor metastasis (Pascual et al., 2017). MSX1 (ENSG00000163132) encodes a member of the muscle segment homeobox gene family, and the overexpression of MXS1 can inhibit the proliferation of tumor cells (Lu et al., 2016). MSX1 could regulate the Wnt/β-catenin pathway in glioblastoma, thereby inhibiting the migration and invasion of cancer cells (Tao et al., 2016). In addition, it has been reported that MSX1 and P53 can induce autophagy in cancer cells, thereby inhibiting the occurrence of cancer (Park et al., 2005).

Further improvement is still needed in the model constructed in this study. The combination of mRNA prognostic biomarkers was based on the information of bladder urothelial carcinoma patients in the TCGA dataset, and it was verified only in the TCGA validation set and two GEO datasets. To further confirm the applicability of the model, more bladder urothelial carcinoma data were needed to be accumulated in the future. At the same time, the effect of these eight mRNA biomarkers in improving the quality of life in patients was still needed to be verified in clinical practice. In addition, the underlying mechanism needs to be further studied. Among the eight genes contained in the model, only three genes were reported to be related to cancer, while the other five genes have not yet been reported to be related to the development of cancer. On one hand, this is probably due to the limitations of current research, and the lack of evidence linking these five genes directly to cancer. On the other hand, it might also be that these genes are not directly related to cancer, and changes in their expressions are only indirectly affected by the metabolic abnormalities caused by cancer. Because of the lack of these information and data at present, the function of the five genes in the model and their association with cancer are not clear yet, and thus it is difficult to determine the accuracy of the above speculations, which need to be addressed by future studies.

## Conclusions

In conclusion, we constructed eight mRNA biomarker model for the prognosis of bladder urothelial carcinoma. This model has high accuracy and good applicability in both TCGA dataset and two cross-platform GEO datasets. When compared with the predictive performance of molecular prognostic markers found in other studies, the mRNA prognostic biomarker combination model also shows significant advantages, which indicates that the model has a certain practical value. Therefore, the risk stratification of patients by using this model is expected to provide a reference for the selection of clinical treatment strategies in the future.

## Supplemental Information

10.7717/peerj.7836/supp-1 Supplemental Information 1 Figures S1–S5.

10.7717/peerj.7836/supp-2 Supplemental Information 2 Dataset S1.

10.7717/peerj.7836/supp-3 Supplemental Information 3 Dataset S2.

10.7717/peerj.7836/supp-4 Supplemental Information 4 Dataset S3.

## Additional Information and Declarations

### Data Availability

The following information was supplied regarding data availability: We downloaded the data from publicly available databases: The Cancer Genome Atlas database (https://cancergenome.nih.gov/; accession ID TCGA-BLCA) and the GEO database (accession numbers GSE13507 and GSE31684).
