# Supplementary material for: An eight-mRNA signature predicts the prognosis of patients with bladder urothelial carcinoma
Source: PeerJ. 2019 Oct 22;7:e7836. doi: 10.7717/peerj.7836 (PMC6814068; doi:10.7717/peerj.7836)
Supplement: Supplemental Information 1 [file peerj-07-7836-s001.docx]

Supplementary Material:


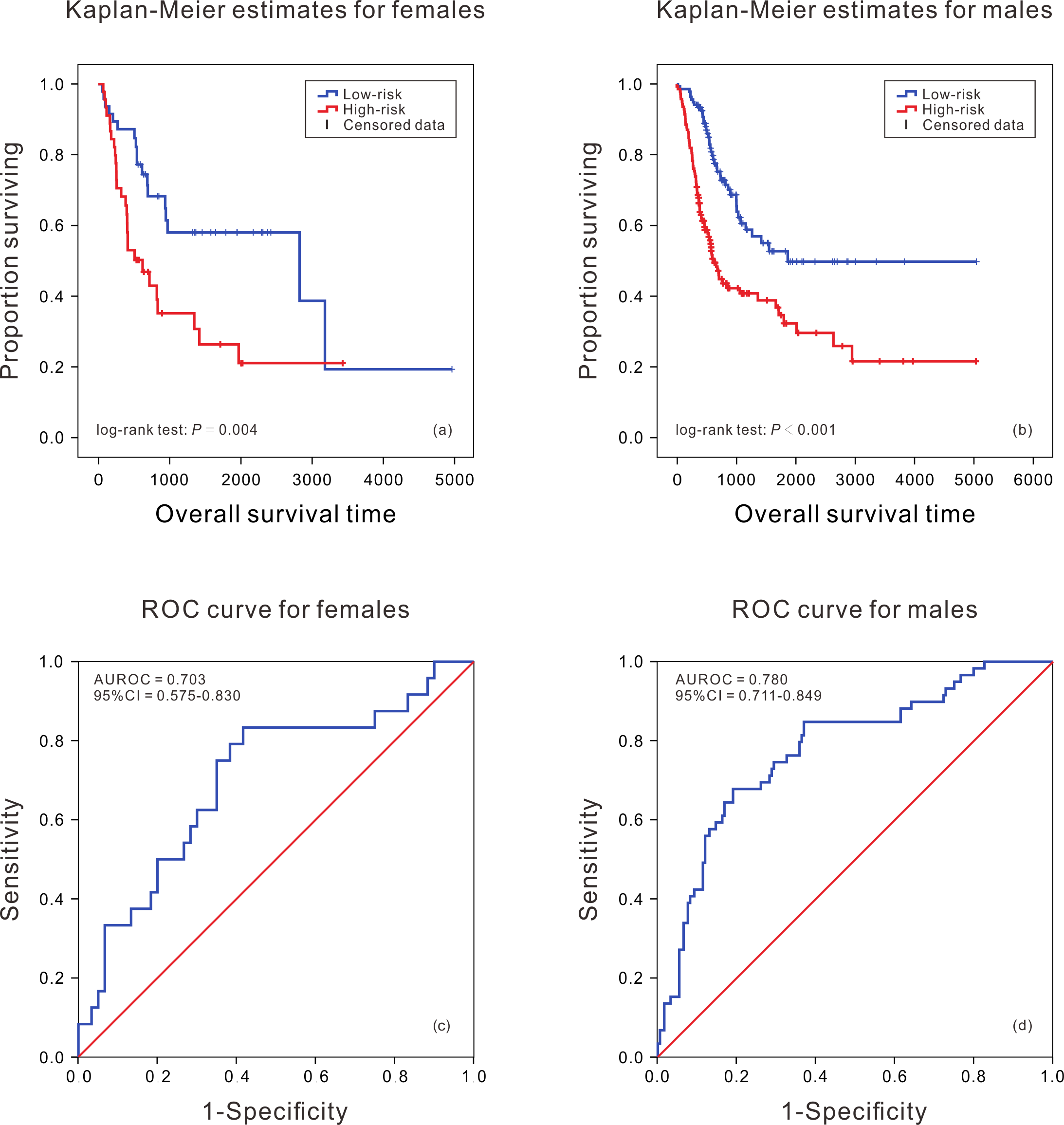


**Figure S1.** Kaplan-Meier analysis of survival difference between high and low risk BLCA patients and ROC curve of survival prediction by RNA prognostic biomarker in different gender groups.


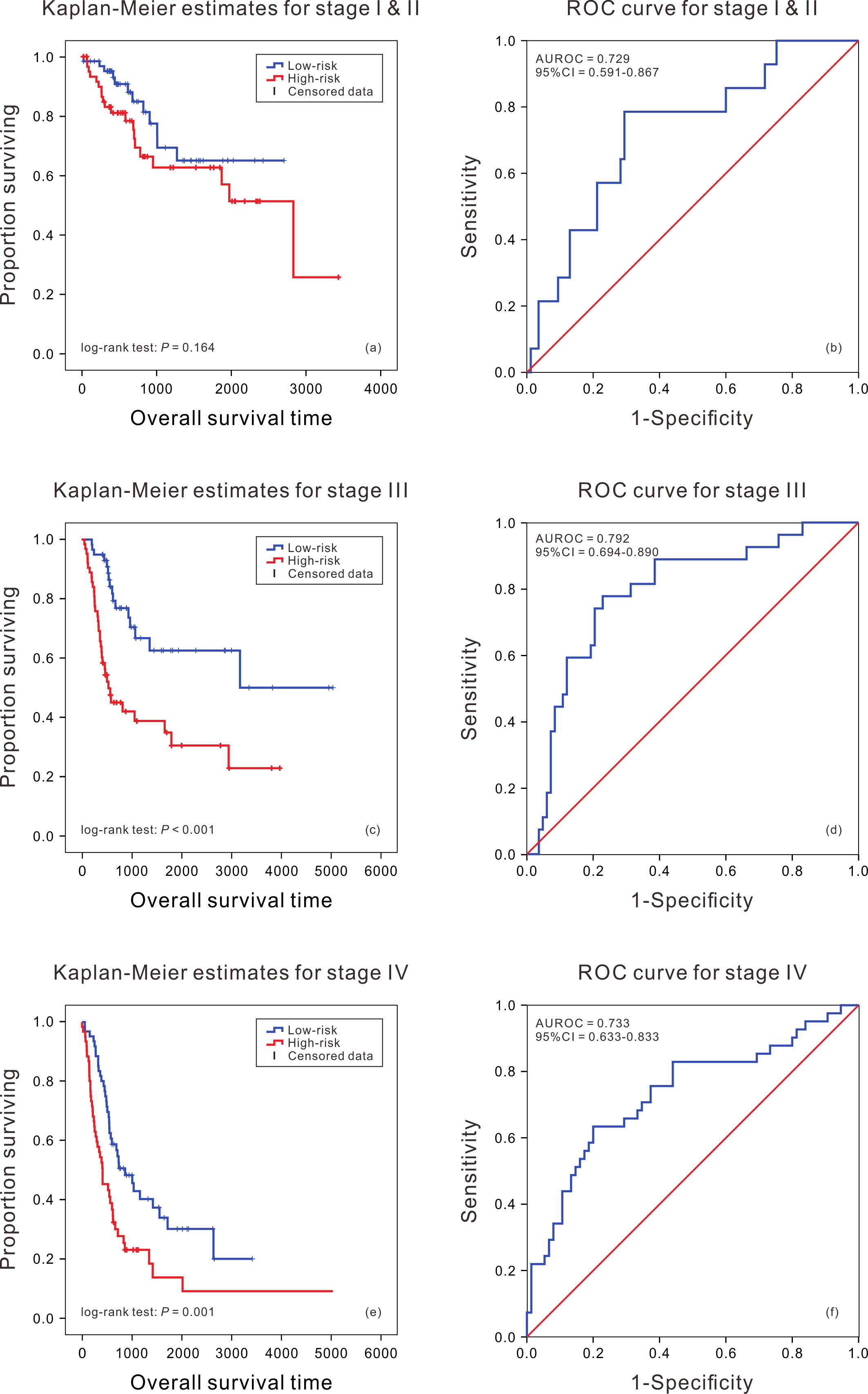


**Figure S2**. Kaplan-Meier analysis of survival difference between high and low risk BLCA patients and ROC curve of survival prediction by RNA prognostic biomarker in different clinical stages.


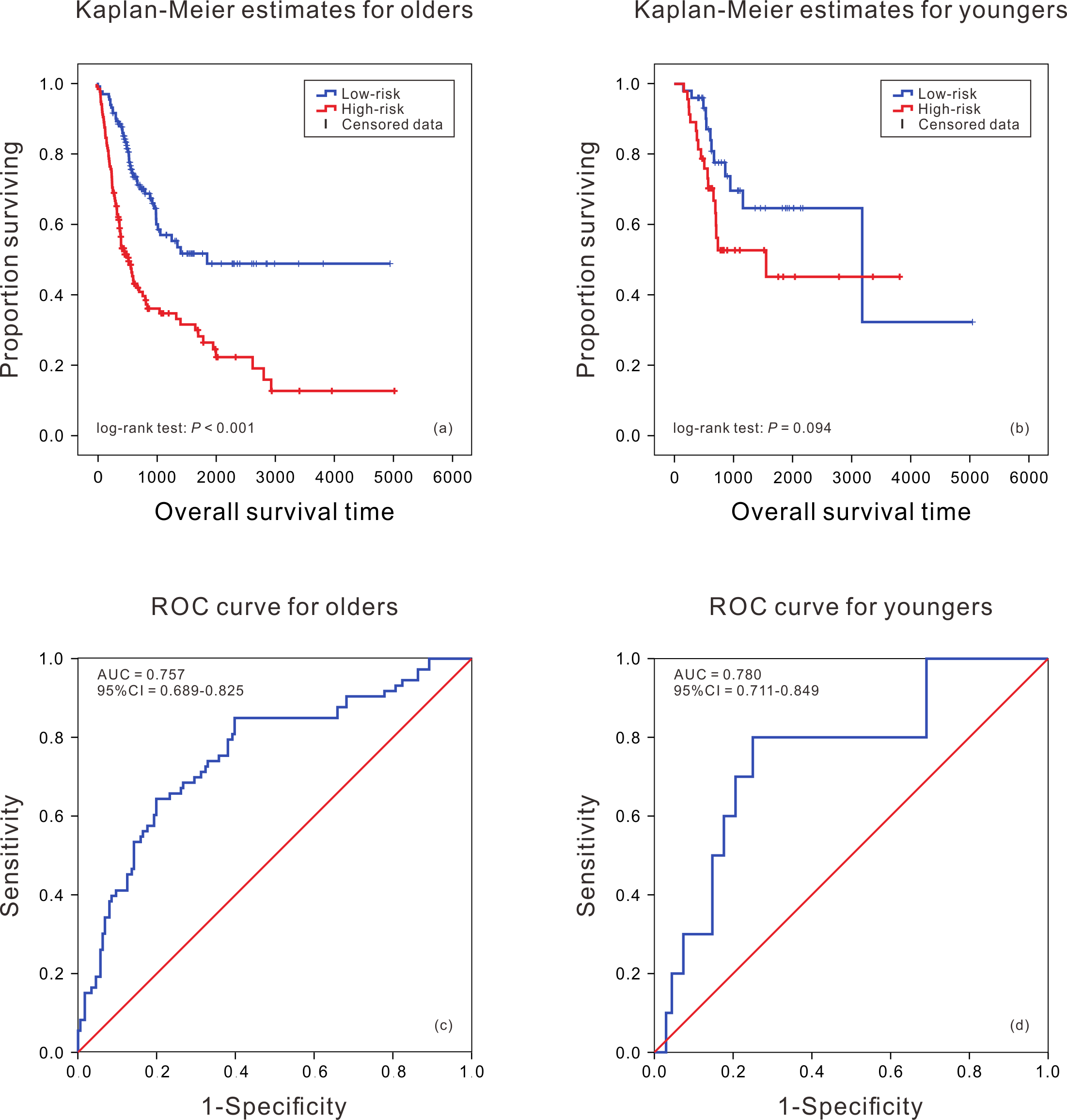


**Figure S3**. Kaplan-Meier analysis of survival difference between high and low risk BLCA patients and ROC curve of survival prediction by RNA prognostic biomarker in different age groups.


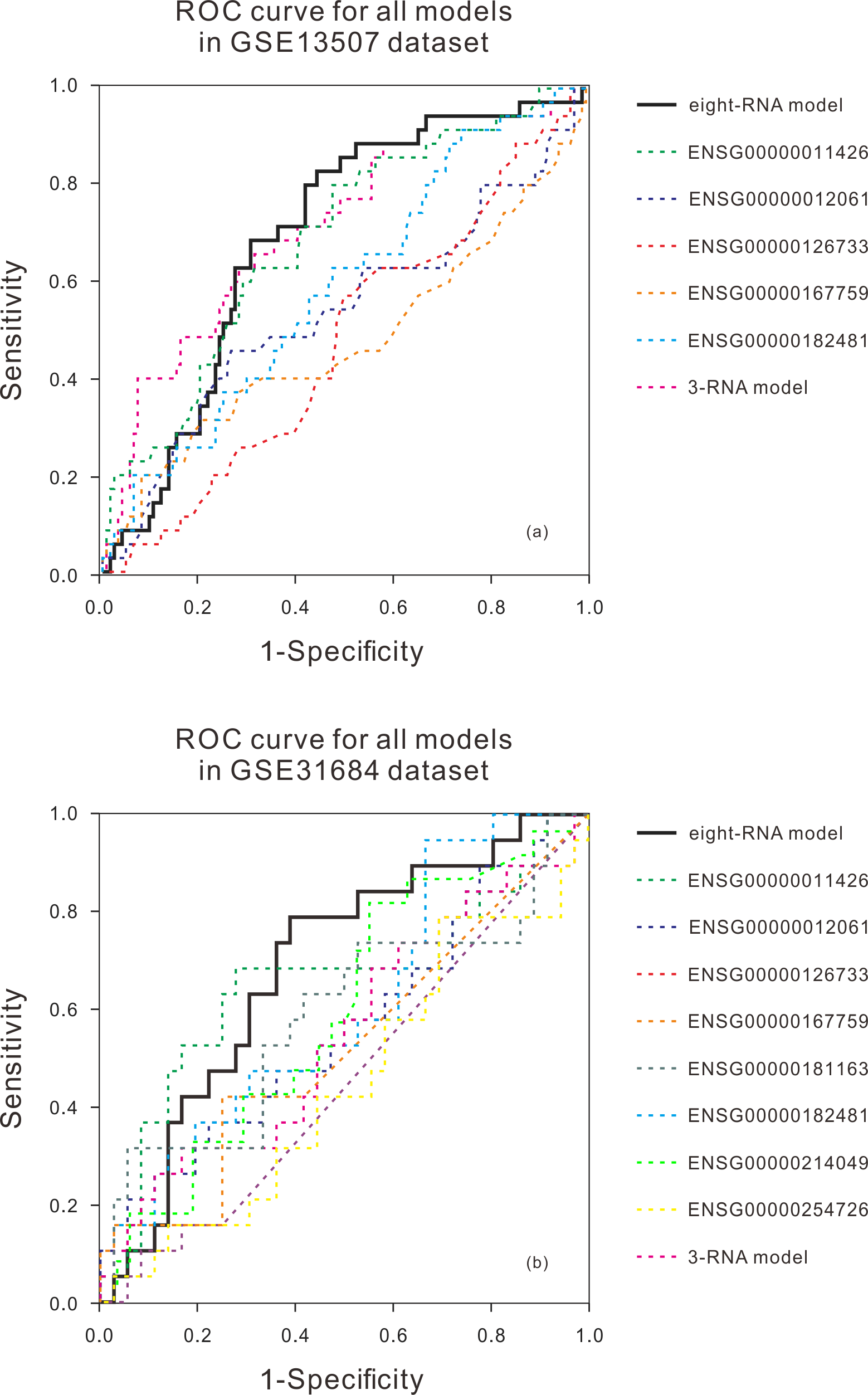


**Figure S4.** The ROC curve of prediction property of RNA prognostic biomarker combination for BLCA in comparison with other known prognostic biomarkers in GSE13507 dataset (a) and GSE31684 dataset (b).


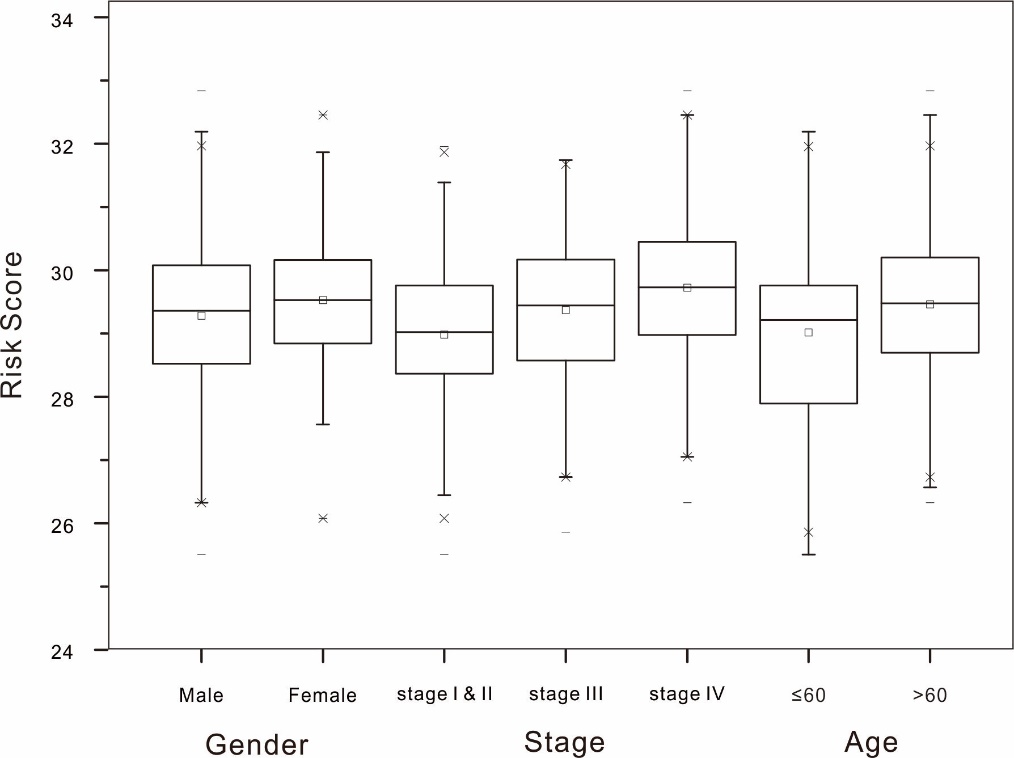


**Figure S5.** The boxplot of risk scores for different genders, cancer stages and ages.
